# Molecular genetic study in hepatocellular carcinoma: microRNA deregulation in multistep hepatocarcinogenesis

**DOI:** 10.1186/1479-5876-10-S2-A9

**Published:** 2012-10-17

**Authors:** Irene Oi-Lin Ng

**Affiliations:** 1 State Key Laboratory for Liver Research and Department of Pathology, the University of Hong Kong, Hongkong, China

## 

Deregulation of microRNAs (miRNAs) plays an important role in human carcinogenesis. However, miRNA deregulation in the pre-malignant lesions and expression changes during multistep hepatocarcinogenesis remain elusive. We investigated the expression changes of seven cancer-related miRNAs during the early stages of HBV-related hepatocarcinogenesis, including dysplastic nodules (DN), small hepatocellular carcinomas (HCCs), and their corresponding non-tumorous livers. We found that down-regulation of miR-145 and miR-199b and up-regulation of miR-224 were frequently observed in pre-malignant DNs and these changes persisted throughout HCC development. Restoration of miR-145 in both HepG2 and Hep3B HCC cells significantly inhibited cell proliferation and reduced cell migration and cell invasion. Furthermore, these inhibitory functions of miR-145 could be substantially reduced by anti-miR-145 inhibitor. Our results showed that miRNA deregulation was an early event and accumulated throughout the various steps of HBV-associated hepatocarcinogenesis.

On the other hand, we investigated the mechanisms of HCC metastasis and identified an antimetastatic miRNA, miR-139, that is down-regulated in human HCC samples. Down-regulation of miR-139 in HCC was associated with poor prognosis of patients and features of metastatic tumors, including venous invasion, microsatellite formation, absence of tumor encapsulation, and reduced differentiation. miR-139 expression was reduced in metastatic HCC tumors as compared with primary tumors. Overexpression of miR-139 in HCC cells significantly reduced cell migration and invasion in vitro and the incidence and severity of lung metastasis from orthotopic liver tumors in mice. miR-139 interacted with the 3’ untranslated region of Rho-kinase 2 (ROCK2) and reduced its expression in HCC cells. Levels of miR-139 correlated inversely with ROCK2 protein in human HCC samples. Expanding insight into the keys of miRNA dysregulation involved in HCC metastasis will yield important clues to our understanding of the complicated mechanisms underlying HCC progression and may enhance the development of new therapeutic regimens in treating advanced HCCs.

Presence of tumor thrombi in the portal veins (venous metastases) is a clinicopathological feature of metastatic HCCs. By analyzing the miRNA expression profiles of non-tumorous livers, primary HCCs, and venous metastases in the same livers from 20 HCC patients with low density microarray (LDA), we identified the precise alterations of miRNA expression from non-tumorous livers to primary HCCs and venous metastases globally. Non-tumorous livers were distinctly segregated from primary HCCs and venous metastases, whereas no discernible difference in the expression pattern could be found between primary HCCs and venous metastases. However, a marked global reduction of miRNA expression levels was detected in venous metastases, as compared with primary HCCs. These data suggest that miRNA deregulation may be an early event in liver carcinogenesis and the later global miRNA down-regulation aggravates the pre-existing miRNA deregulation to further promote HCC metastasis. Our study has enriched the current understanding of the deregulation of miRNAs in HCC progression and highlighted the sequential and distinctive alterations of miRNA expression in primary HCC and venous metastasis formation.

